# Sequential displacement of Type VI Secretion System effector genes leads to evolution of diverse immunity gene arrays in *Vibrio cholerae*

**DOI:** 10.1038/srep45133

**Published:** 2017-03-22

**Authors:** Paul C. Kirchberger, Daniel Unterweger, Daniele Provenzano, Stefan Pukatzki, Yan Boucher

**Affiliations:** 1 Department of Biological Sciences, University of Alberta, Alberta, Canada; 2 Department of Zoology, University of Oxford, Oxford, United Kingdom; 3 Department of Biological Sciences, University of Texas Rio Grande Valley, Texas, United States; 4 Department of Immunology & Microbiology, University of Colorado School of Medicine, Colorado, United States

## Abstract

Type VI secretion systems (T6SS) enable bacteria to engage neighboring cells in contact-dependent competition. In *Vibrio cholerae*, three chromosomal clusters each encode a pair of effector and immunity genes downstream of those encoding the T6SS structural machinery for effector delivery. Different combinations of effector-immunity proteins lead to competition between strains of *V. cholerae*, which are thought to be protected only from the toxicity of their own effectors. Screening of all publically available *V. cholerae* genomes showed that numerous strains possess long arrays of orphan immunity genes encoded in the 3′ region of their T6SS clusters. Phylogenetic analysis reveals that these genes are highly similar to those found in the effector-immunity pairs of other strains, indicating acquisition by horizontal gene transfer. Extensive genomic comparisons also suggest that successive addition of effector-immunity gene pairs replaces ancestral effectors, yet retains the cognate immunity genes. The retention of old immunity genes perhaps provides protection against nearby kin bacteria in which the old effector was not replaced. This mechanism, combined with frequent homologous recombination, is likely responsible for the high diversity of T6SS effector-immunity gene profiles observed for *V. cholera*e and closely related species.

The family Vibrionaceae consists of over 100 related species of highly motile, heterotrophic bacteria that enzymatically convert inaccessible organic matter found in aquatic environments into carbon sources available to higher trophic levels of the ecosystem they inhabit^1^. Numerous mostly harmless lineages of *Vibrio* coexist within niches, competing for largely similar resources^2^. Among them are a few human pathogens of relevance, including *Vibrio cholerae,* the causative agent of the sometimes dramatic and lethal cholera diarrhea. More specifically, a single lineage of the *V. cholerae* species, comprised primarily of O1 and O139 serogroup strains^3^, has adapted to effectively colonize the human gastrointestinal tract and is responsible for all known cholera pandemics^4^. Pandemic *V. cholerae* strains harbor the horizontally acquired genetic elements VPI-1 and CTX-Φ, encoding the toxin co-regulated pilus and cholera toxin respectively. These virulence factors enable pandemic strains to colonize the crypts of villi in the small intestine, causing watery purges of diarrhea and releasing billions of pathogenic bacteria into the environment^5^. Thus, pathogenic *V. cholerae* lead a dual lifestyle: One that requires the ability to pursue, attach and colonize biotic surfaces in a relatively oligotrophic aquatic environment of low osmolarity, and another that requires the successful colonization of a eutrophic, biochemically challenging human intestine populated by a highly diverse commensal host flora^6^.

In both of these competitive environments, *V. cholerae* is believed to actively employ their Type VI secretion system (T6SS), which is induced by chitin in the environment^7^ and by bile salts in the gut^8^. The T6SS is a membrane-spanning nanomachine capable of injecting toxin-tipped protein spears into adjacent eukaryotic and bacterial target cells^9^,^10^. The T6SS spear consists of Hcp multimers tipped by a VgrG (hetero)trimer and effector proteins with varying cytotoxic effects^11^. For example, the VgrG-1 protein of some *V. cholerae* strains harbors a C-terminal domain that mediates crosslinking of cytoskeletal actin fibers in eukaryotic cells (such as predatory amoebae or macrophages), leading to cell rounding and death^12^,^13^. VgrG-3, on the other hand, displays antibacterial properties by degrading prokaryotic peptidoglycan, and is also an important factor in the colonization of the human intestine^14^,^15^. Additionally, so-called cargo effectors can be loaded onto the Hcp-VgrG spear, further expanding the toxic capabilities of the T6SS^16^.

Unterweger *et al*.^17^ found that a multitude of diverse T6SS effector-immunity (EI) gene modules are encoded in different *V. cholerae* genomes. Effector proteins are placed as cargo onto the T6SS-spear by an adaptor protein, while immunity proteins remain inside the cell and prevent intoxication by incoming cognate effectors^18^. The resulting “poisoned” spear proves lethal to target cells that do not possess an EI module of the same type^17^. Through this system, strains of *V. cholerae* are not only able to attack eukaryotes and bacteria belonging to different species, but also their perhaps strongest competitors, non-kin strains of the same species^19^. Unterweger *et al*.^17^ established a three-letter system for typing *V. cholerae* T6SS variants based on their EI modules. Different letters designate unique EI gene families (as defined by a 30% amino acid identity of immunity proteins) encoded in three genomic clusters: aux-1 (A and C), aux-2 (A-E) and the large cluster (A-G). In the case of the large cluster, the effector is a domain at the 3′ end of *vgrG-3,* not a separate gene (Fig. 1). Strains with identical EI module composition belong to the same compatibility groups, whereas those that possess different EI modules are T6SS incompatible. For example, most *V. cholerae* from the lineage containing pandemic strains belong to the AAA type (note that the same letter for different clusters does not denote the same gene family) and can co-exist among each other (they are “compatible”). In contrast, strains of the AAA-type engage in T6SS-mediated competitive interactions with strains of different groups such as CAG or AAC, and are thus “incompatible” with them^17^. The AAA genotype appears to be the most effective for intraspecies competition under laboratory conditions, as suggested by the ability of *V. cholerae* V52 (an O37 serogroup strain from the same lineage as O1 serogroup pandemic strains) to outcompete any strain with a different EI module combination^17^. In addition to each *V. cholerae* strain possessing three EI modules at conserved genomic clusters, recent studies have found further EI modules encoded on horizontally transferred genomic islands that potentially expand the T6SS mediated competitive abilities of *V. cholerae* even further^20^,^21^.

The large number of distinct EI modules may indicate an ongoing evolutionary arms race to succeed in intra- and interspecies competition and to overcome eukaryotic host defenses. Such intense selective pressure could facilitate not only rapid mutational divergence of effectors and immunity proteins of different lineages, but also horizontal gene transfer, either as a whole or in parts, giving rise to new variants.

To elucidate the evolutionary dynamics of the *V. cholerae* T6SS, we performed a systematic survey of the T6SS-harboring genomic regions in *V. cholerae* and its closest relatives among the Vibrionaceae. This led to the discovery of additional putative effector and immunity genes in the 3′-region of several *Vibrio* T6SS clusters. Additionally, we find evidence that insertion of distinct EI modules replaces old effectors, yet often retains the immunity genes, leading to an array of multiple different orphan immunity genes and the establishment of new types of mosaic T6SS regions. We also provide evidence that such modular insertion may have given rise to the unique combination of effector and immunity genes found in pandemic *V. cholerae* strains.

## Material and Methods

### Identification and annotation of T6SS clusters in Vibrio species

Initial screening for T6SS clusters in *Vibrio cholerae* and the closely related *Vibrio metoecus, Vibrio mimicus, Vibrio fluvialis*, and *Vibrio furnissii* was conducted by performing megaBLAST searches against all genomes of these species (*V. cholerae*: 548, *V. metoecus*: 10, *V. mimicus*: 10, *V. fluvialis*: 8, *V. furnissii*: 4) available on NCBI. Genes VC1421, VCA0022 and VCA0125 of *V. cholerae* strain N16961, each located downstream of one of the three T6SS clusters, were used as conserved, single copy query sequences to identify contigs containing aux-1, aux-2 and large T6SS cluster genes, respectively. T6SS cluster were then located on the extracted contigs by mapping them against N16961 *vgrG*-1, *vgrG*-2 and *vgrG*-3 (VC1416, VCA0018 and VCA0123) in Geneious 8^22^. Homologous genes within those regions were then identified by extracting all ORFs > 300 bp and conducting all-vs-all local BLASTP searches on translated sequences. Hits with a minimum of 30% protein sequence identity were considered homologous and annotated accordingly. To ensure completeness of our initial genomic survey, all identified putative effector and immunity genes found in our first round of searches were then used as query for a second round of megaBLAST searches, and additional T6SS regions found by these searches were added to the dataset. This dataset was then trimmed to 95 genomes (Supplementary Table 1) by eliminating all genomes that did not show any nucleotide divergence in the identified T6SS clusters. Additional megaBLAST searches for the mobile antibacterial *tseH*-*tseI* EI module^20^ and T6SS cluster identified by Labatte *et al*.^21^ revealed their presence in various *V. cholerae* strains.

### Phylogenetic analysis

From the finalized selection of genomes, a pangenome k-mer SNP dataset was extracted using kSNP3.0 with an inferred optimal k-mer size of 19^23^. This dataset contained a set of 1,085,207 19-mers found in at least one genome (with absences in other genomes denoted as missing data).

A maximum-likelihood phylogenetic tree was then calculated using RAxML 8.0 using the GTRGAMMA substitution model, and statistical support was estimated based on 100 bootstrap replicates^24^. This whole-genome rather than core-genome approach provides increased phylogenetic resolution when comparing a large number of closely related genomes (belonging to a single species) while also including more distantly related genomes in the dataset. In a core-genome approach, the inclusion of more distantly related genomes or incomplete draft genomes (only a small number of *Vibrio* genomes are complete) leads to a loss of resolution since phylogenetically informative characters needed for the differentiation of closely related genomes are removed due to their absence in some genomes. The whole genome approach becomes more problematic over longer evolutionary timeframes where the cumulative effect of horizontal gene transfer might trump the higher incidence of vertical descent in closely related genomes^25^. To avoid potentially false inference of relationships in more ancestral branches of the tree, we collapsed bipartitions with bootstrap support lower than 70.

Alignments of single genes or gene regions were performed using the CLUSTALW^26^ plugin in Geneious 8 using standard settings and then manually edited in Geneious 8. Phylogenetic trees were generated from these alignments using RAxML as described above.

### Recombination analysis

In order to infer putative regions within T6SS clusters that have undergone horizontal gene transfer, recombination analysis was performed using RDP4^27^. Four different algorithms implemented in RDP4 were used: GENECONV, RDP, MAXCHI and CHIMAERA. Briefly, GENECONV^28^ identifies regions of sequence pairs in an alignment with significantly lowered amount of nucleotide polymorphisms compared to the whole region. The RDP algorithm^27^ performs a sliding window analysis along triplet sequences in an alignment and identifies regions of high sequence similarity incongruent with an UPGMA dendrogram created from the entire alignment. MAXCHI^29^ and (in a modified form) CHIMAERA^30^ identify putative recombination breakpoints by moving a bi-partitioned sliding-window along a sequence pair and detecting significant differences in sequence similarity between the two sides of the window.

Since different algorithms do not always identify the same recombination events, only events detected by at least three out of four algorithms were counted as valid. Detection of regions affected by homologous recombination and identification of recombination breakpoints was conducted on alignments of each cluster type separately (i.e. an alignment of A-type aux-1 clusters), as the presence of large non-homologous alignment regions impairs correct identification. For the same reason, regions of the same type that contained additional genes, such as multiple copies of immunity proteins that are not present in the majority of sequences, were also left out.

### Whole genome sequencing and assembly

Isolation and DNA extraction of *V. cholerae* strains DL4211 and DL4215 from the Rio Grande estuary was described in a previous study^19^. Whole genome sequencing was performed by Ambry Genetics (CA, USA) using 100 bp paired-end Illumina HiSeq 2000 technology after following the TruSeq DNA sample preparation guidelines. De-novo assembly of reads into contiguous sequences was then conducted using CLC Genomics Workbench 5.0 (CLC Bio, Aarhus, Denmark). The two draft genomes were submitted to NCBI GenBank and given the accession numbers MOLL00000000.1 (DL4211) and MOLM00000000.1 (DL4215).

## Results and Discussion

### T6SS cluster structure is conserved in *V. cholerae* and its closest relatives

**Figure 1:**
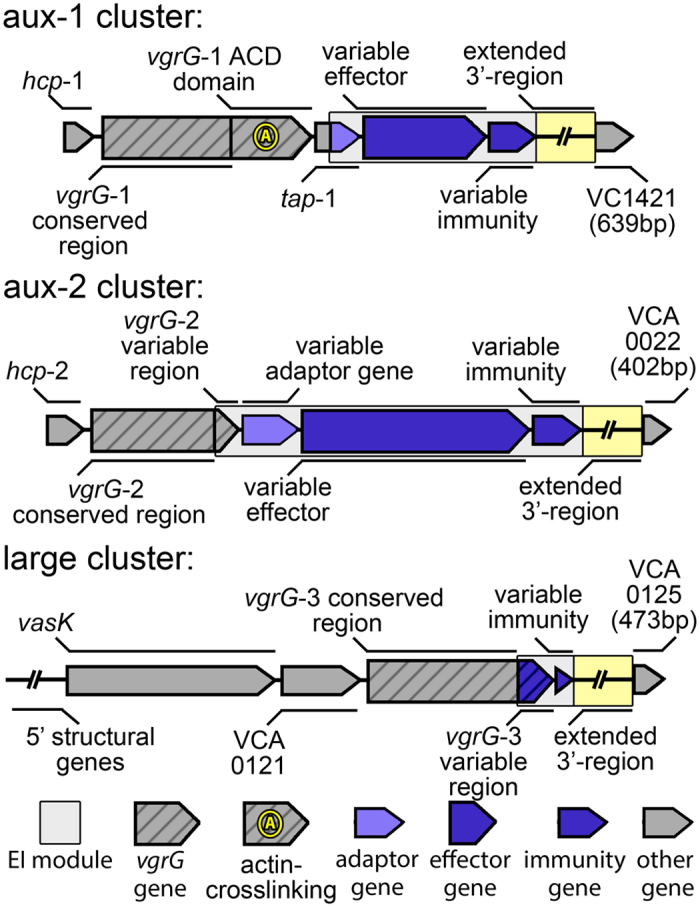
Schematic organization of *V. cholerae* T6SS clusters. Striped arrows denote genes encoding VgrG effectors; non-striped coloured arrows denote variable effector (large arrows) or immunity genes (small arrows); grey arrows indicate conserved genes based on cluster tags from the reference genome of *V. cholerae* N16961. Not pictured: VCA0122, the coding region of which is frequently interrupted by deletions. Boxed region denotes the EI module. Extended 3′-region is of variable length and gene content, and all coloured genes vary in length. Large cluster extends further upstream than shown in figure.

**Figure 2:**
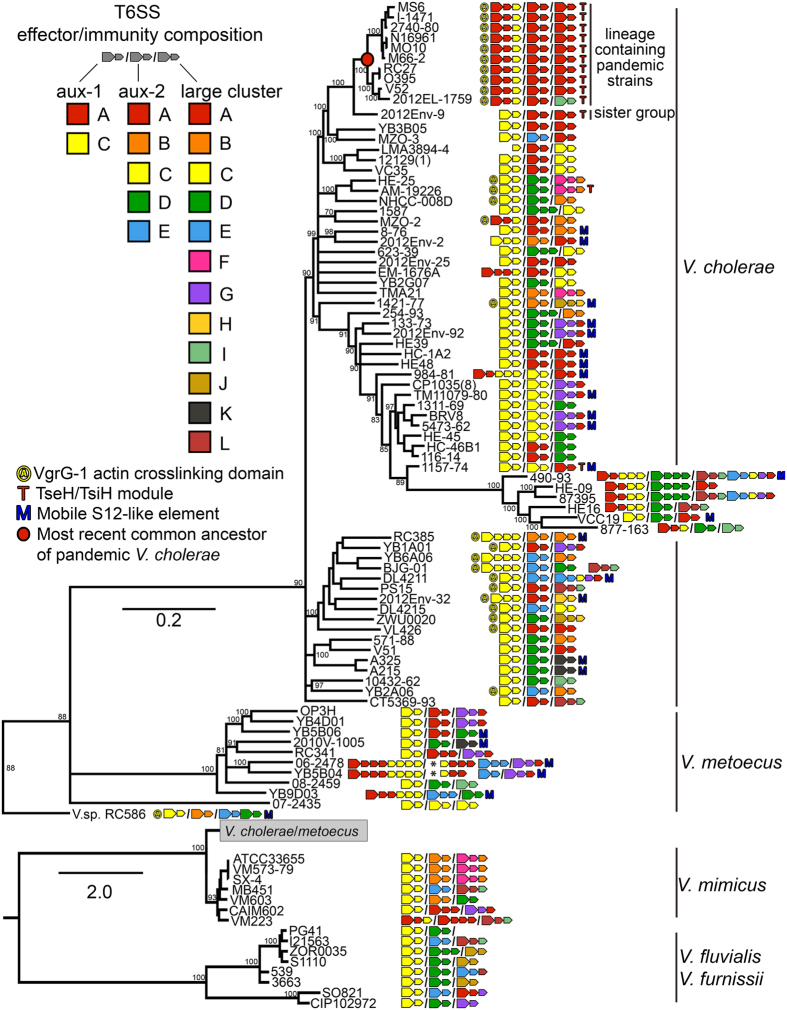
Whole-genome phylogeny and T6SS EI module composition of *Vibrio cholerae* and closely related species. Large arrows next to strain names indicate effector, small arrows immunity genes. Auxiliary cluster 1, 2 and the large cluster are separated by slashes. Asterisks indicate transposons. The phylogenetic tree was calculated using the GTR + Gamma Maximum likelihood model implemented in RAxML based on a 1,085,207 pangenome 19-mer alignment (including not just characters shared by all, but by at least two genomes) created using kSNP3. *V. cholerae*/*V. metoecus* are visualized separately for better visibility of short internal branches. Statistical branch support was obtained from 100 bootstrap repeats. Bootstrap support for relevant bipartitions is indicated, and branches with support <70 were collapsed. Scale bar indicates substitutions/site. Accession numbers of genomes are listed in Supplementary Table 1.

We identified and annotated T6SS clusters in the publically available genomes of *V. cholerae*, and its four closest relatives *V. metoecus*^31^, *V. mimicus*^32^, *V. furnissii* and *V. fluvialis*^1^. All investigated strains possess the same three clusters structure as previously described for *V. cholerae*^12^ (Figs 1 and 2): one large cluster and two auxiliary clusters (here termed aux-1 and aux-2). The large cluster includes 17 (or 18, depending on the presence of the regulator protein VCA0122^10^) structural genes that encode proteins forming the membrane-spanning machinery of the T6SS, which also contains the *vgrG*-3 gene and an immunity gene encoding a protein protecting against the antibacterial activity of the VgrG-3 C-terminus^15^,^33^. The aux-1 and aux-2 clusters share a common structure: an allele encoding the secreted Hcp protein, a VgrG protein (VgrG-1 and VgrG-2 in aux-1 and aux-2, respectively) followed by an adaptor protein, an additional effector and a cognate immunity protein^17^ (Fig. 1). VgrG-2 proteins, previously described as differing from VgrG-1 and VgrG-3 due to their lack of variable C-terminus^34^, were found to also encode a variable region of around 60 amino acids in length. Although no known functional domain was identified in this region, the sequence of this variable C-terminus varies considerably between strains carrying different EI module types at the aux-2 cluster (with the exception of A and B-types, which carry similar C-termini) (Fig. S1a). Each specific combination of VgrG-2 C-terminus and effector-immunity protein pair is also accompanied by a specific putative adaptor protein (Fig. S2). In light of the functional link of the 3′-end of the aux-1 Tap*-*1 protein with cargo effectors^18^ as well as the aux-2 A-type adaptor gene *vasW* with the A-type effector *vasX*^35^, it is possible that both the VgrG-2 variable C-terminus and effector-specific adaptor proteins encoded in aux-2 are involved in the loading of effector proteins onto VgrG-2. For this reason, we include them in our definition of an EI module (Fig. 1).

**Figure 3:**
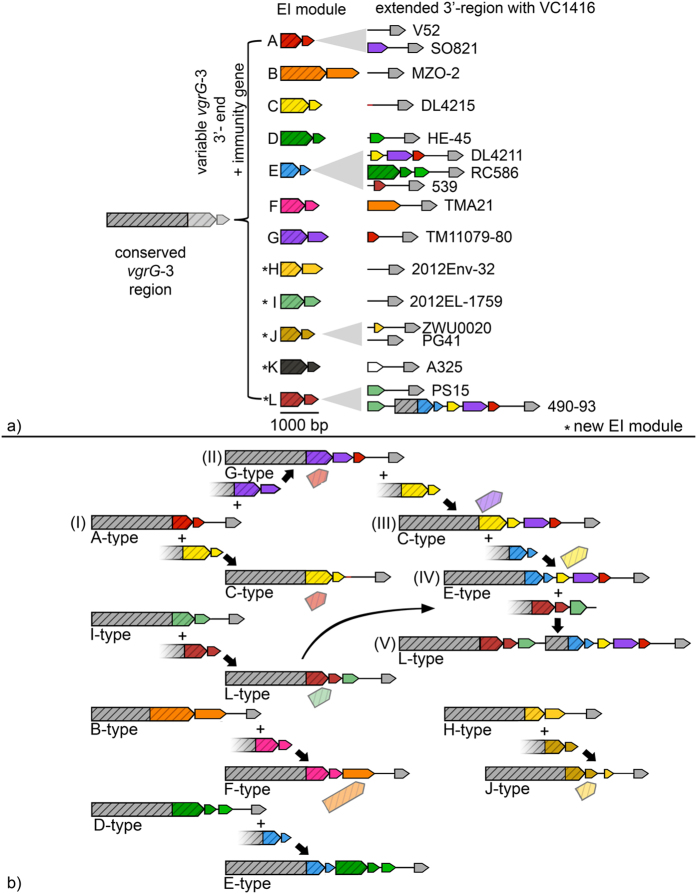
Organization and evolution of the *Vibrio cholerae* large T6SS cluster EI modules. (**a**) Large cluster EI module organization and variability. Conserved 5′-region of *vgrG*-3 can be tipped with different effector encoding 3′-regions and cognate immunity genes. (**b**) Recombinatorial reshuffling of *vgrG*-3. Cluster evolution proceeds by insertion of EI module and variable length of conserved *vgrG*-3 region into ancestral *vgrG*-3 gene. New EI module often (but not always) replaces original effector and shifts immunity gene(s) downstream. Integration of more complex EI clusters can result in larger clusters containing multiple EI components. Arrows indicate coding sequences, lines noncoding regions. Striped arrows denote genes encoding VgrG-3 effectors; non-striped arrows depict variable effector (large arrows) or immunity genes (small arrows). Colours indicate homology between either effector or immunity genes. Identically coloured effector and immunity genes are part of the same EI module. Grey arrows indicate conserved genes. Exact genomic locations can be found in Supplementary Table 2.

We also discovered five novel variable 3′-ends of *vgrG*-3 encoding putative effector domains associated with the large T6SS cluster, along with their corresponding immunity protein coding genes (Fig. 3a and Fig. S1b). In accordance with Unterweger *et al*.’s nomenclature^17^, these were named H-L, following the previously described A-G types. Only two of the novel effector domains corresponded to known proteins or could be assigned a putative function. The I-type effector contains a DUF3380/pfam11860 domain, which is annotated as a phage-derived peptidoglycan binding/muraminidase protein. The K-type effector contains a lambda phage derived lysozyme (cd00736/COG4678).

### Multiple additional immunity genes can be present downstream of effector-immunity modules

The regions between the canonical EI modules and the conserved genes VC1421, VCA0022 and VCA0125 (downstream of aux-1, aux-2 and the large cluster, respectively) vary considerably in number and type of genes between closely related strains (Fig. 2). Parts of these extended 3′-regions are homologous to previously described immunity genes, but not necessarily to those corresponding to the strain’s cognate effector protein (Fig. 3a). In other words, the regions downstream of the EI module in each T6SS cluster in many cases appear to consist of arrays of alternate immunity genes that could cumulatively confer not only resistance to a strain’s own, but also to a number of different additional effectors.

The aux-1 cluster of *V. cholerae* may harbor one of two types of EI modules, either the A or C. In our analysis, the EI module previously identified as a B-type in strain LMA3894-4^17^ appears to be a divergent C-type resulting from a fusion of the *vgrG*-1 and C-immunity gene, lacking an effector. While some strains contain up to three C-type immunity genes (Fig. 2), their presence is independent of whether the strain contains an A or C-type effector gene. C-type immunity genes are in fact universal at the aux-1 cluster, either as part of the strain’s EI module or their extended 3′ region. *V. metoecus*, thus far isolated exclusively from North American coastal environments and a small number of blood and stool samples from the United States^31^, harbors multiple C-type and up to three A-type immunity genes. In some *V. metoecus* genomes, the total number of A- and C-type immunity genes in the aux-1 cluster can be as high as seven (Fig. 2).

In the aux-2 cluster of *V. cholerae*, for which five EI pairs have been described (A-E), the number of immunity genes varies only for the D-type immunity protein. Similar to the aux-1 cluster, most genomes contain just a single aux-2 immunity gene (matching the effector found upstream), but several strains contain up to three. Additionally, *V. mimicus* strain CAIM602 possesses two A-type immunity proteins and *V. mimicus* VM223 harbours three. The most complex arrays are again observed in *V. metoecus*. The aux-2 region in two strains (YB5B04 and 06-2478, also containing seven immunity genes in the aux-1 cluster) appears to be disrupted by a transposon insertion: the extended 3′-region containing A- and C-type immunity genes and further genes typically found downstream of their E-type EI module are located in the vicinity of a transposase in a different region of their genomes (Fig. 2).

The T6SS large cluster, like the auxiliary clusters, can contain additional immunity genes of a different type than the one found in the canonical EI module. In a few instances, effectors matching these additional immunity genes are also found in the extended 3′-region (Fig. 3a). For example, the closely related *V. cholerae* isolates 87395 and 490-93 harbor an L-type EI module, followed by an extended 3′-region containing an I-type immunity gene, a short conserved region usually found downstream of normal effectors, a partial E-type *vgrG-3* effector gene, the cognate E-type immunity gene, a C-type immunity gene, a G-type immunity gene, and finally an A-type immunity gene (Fig. 3).

### Horizontal gene transfer of effector-immunity modules

Like in Proteobacteria in general^36^, effector and immunity gene distribution mapped on the phylogeny of *Vibrio* shows both patterns of vertical inheritance as well as horizontal gene transfer (HGT) (Fig. 2). The vast majority of aux-1 EI-modules in non-pandemic strains of *V. cholerae* are of the previously described C-type^17^. Despite being rare in *V. cholerae* overall, aux-1 A-type EI modules are ubiquitous in the *V. cholerae* clade containing pandemic strains as well as a divergent clade containing strains 490-93 and 877-163, indicating independent acquisitions in the ancestors of these two clades and subsequent vertical inheritance (Fig. 2). A few strains outside of these clades, as well as strains of *V. metoecus* and *V. mimicus,* also possess this type of module. The VgrG-1 protein containing an actin-crosslinking domain also displays a distribution pattern indicative of horizontal gene transfer: It is only found in few divergent lineages of *V. cholerae* (including the lineage containing pandemic strains) while virtually absent in *V. metoecus, V. mimicus, V. furnissii* and *V. fluvialis* (Fig. 2). Instead, a truncated version of VgrG-1 is present in most investigated strains. The truncated protein is fully functional in its antibiotic activity^18^, yet lacks the C-terminal actin-crosslinking domain that is involved in cytotoxicity against human macrophages and predatory *Dictyostelium* slime molds^12^. This disparate distribution of the A-type EI module as well as the VgrG-1 actin-crosslinking domain suggests that they were, like the major *V. cholerae* virulence factors *tcp* or *ctx*^37^,^38^, independently introduced into various lineages by horizontal gene transfer.

Aux-2 EI modules C and E as well as large cluster modules C, D, F and G show similar phylogenetically disparate distributions. For example, the aux-2 C-type and the large cluster D-type EI modules are predominantly found in a single, well-supported clade of *V. cholerae* (containing among others the atypical O1 serogroup strain TM11079-80) as well as occasionally in distantly related genomes (Fig. 2). HGT is also apparent in single gene phylogenies of effector and immunity genes (Figs S4–S7): alleles from closely related strains often fall into different gene clusters.

Disparate distributions are also found for additional recently discovered EI modules located outside of the three known *Vibrio* T6SS clusters. The *tseH*-*tseI* EI module previously described by Altindis *et al*.^20^ rarely appears outside the *V. cholerae* pandemic group (Fig. 2). This EI module is located 3.5 kb upstream of the chromosomal integron region of *V. cholerae* and possesses antibacterial activity^20^. An additional auxiliary cluster containing *hcp*, a *vgrG*-allele and what appears to be a novel EI module, was recently found encoded on a genomic island termed GI*Vch*S12^21^. EI modules of the GIVchS12-type are irregularly distributed as well (Fig. 2).

### Homologous recombination without specific integration sites leads to the mosaic structure of T6SS clusters

**Figure 4:**
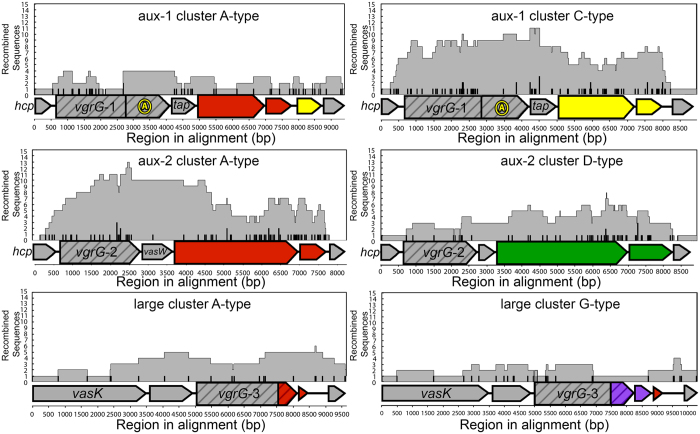
Location of recombination tracts and breakpoint on T6SS clusters. X-axis indicates region in the alignment starting at *hcp*-1 and 2 for aux-1 and aux-2 cluster and *vasK* for large cluster. Grey areas indicate presence of a recombined region detected by >3 algorithms implemented in RDP4, vertical hash marks indicate presence of corresponding recombination breakpoints in that region of the alignment. Multiple recombination regions/breakpoints are stacked on top of each other. Horizontal block arrows indicate coding sequences, horizontal lines noncoding regions. Striped arrows denote genes encoding VgrG effectors; non-striped arrows variable effector (large arrows) or immunity genes (small arrows). Colours indicate homology between either effector or immunity genes. Identically coloured effector and immunity genes are part of the same EI module. Grey arrows indicate conserved genes.

Distribution patterns of EI modules and phylogenies of effector and immunity genes suggest that genes within T6SS clusters are frequently transferred between *Vibrio*. These transfer could occur at random sites, or at specific recombination sites like the integration of the *Vibrio* pathogenicity island VPI-1^39^. In the latter scenario, the size of recombined regions as well as the location of recombination breakpoints should not vary greatly between events, as each recombinant region would be integrated at a specific location. Such recombination hotspots have been predicted to exist in the adaptor protein encoding gene *tap-1* and *vgrG-1* of the aux-1 cluster^18^. We performed a scan for recombinant regions in T6SS clusters based on phylogenetic discordance and patterns of single nucleotide polymorphisms^27^. Contrary to expectations of recombination being more frequent at a specific site, the size of recombined regions and the associated positions of recombination breakpoints varied greatly in all three T6SS clusters (Fig. 4). We could detect a number of recombination breakpoints located around the hypothesized recombination hotspots. However, we also found numerous recombination breakpoints inside effector and immunity genes, structural genes and noncoding regions. Interestingly, we detected very few recombination events within the actin-crosslinking domain at the 3′-end of *vgrG*-1, but found a relatively large number of breakpoints at the 5′-end of this gene. As hypothesized for bacteriocin genes, this difference in the number of detected recombination events could simply be a result of the availability of specific genes or gene regions as substrate for recombination^40^. The actin crosslinking domain, present in only a small subset of *Vibrio,* could thus only rarely recombine compared to the ubiquitous 5′-end of *vgrG*-1 or the highly conserved central region of *tap*-1.

Overall, recombination appears to not be confined to specific regions within the T6SS clusters (Fig. 4). Horizontally transferred DNA integrates in any sufficiently homologous site and thus essentially everywhere along the T6SS region, potentially incorporating non-homologous regions between the integration sites and making each individual region a mosaic composed of DNA from multiple different origins. This mosaic structure is also apparent in phylogenies of individual T6SS genes, with sequences of genes from distantly related strains clustering together, and weak overall bootstrap support due to the existence of genes composed of DNA from multiple distantly related bacteria (Figs S4–S7). This is particularly apparent in widespread genes such as those encoding C-type effector and immunity proteins (Figs S4 and S5).

### A model for the establishment of immunity gene arrays through displacement of effector genes by EI modules

Homologous recombination exclusively cannot explain the existence of numerous arrays of multiple immunity genes, as their formation involves the addition of novel genes with no homology to sequences present in the recipient strain. We propose an event where a horizontally transferred EI module is inserted into a T6SS cluster, displacing the ancestral effector gene or effector gene domain but conserving the ancestral immunity gene, which is shifted downstream of the new EI module.

A putative mechanism to account for these observations could be akin to previously described homology-facilitated illegitimate recombination: a conserved stretch of an incoming DNA element, in this case represented by the upstream region of the EI module, serves as an “anchor” by forming a heteroduplex with a homologous sequence in the target region, thereby facilitating the integration of the non-homologous end through illegitimate recombination^41^,^42^. Multiple successive EI insertions could then give rise to longer arrays of a single effector with multiple immunity genes as those reported here for numerous strains. The mechanism can be illustrated most accurately by examining the structure of the large cluster (L-type) found in *V. cholerae* strains 87395 and 490-93 (Fig. 3b). This particular gene assembly evolved by successive transitions through various simpler intermediate forms, whose structure is present in other strains included in this study. (I) First, an ancestral A-type EI module is replaced by a G-type EI module, replacing the stretch of DNA encoding the A-type *vgrG-*3 effector region with a G-type effector and immunity gene while shifting the A-type immunity gene to the back of the array; (II) This G-type EI module is then replaced by a C-type EI module, shifting the G and A immunity genes further back (note that this particular cluster arrangement is the only one in this sequence yet to be observed in an extant strain); (III) The new C-type EI module is then replaced by an E-type EI module, giving rise to an E-type EI module followed by C, G and A immunity genes; (IV) A final insertion event of an L-type EI module (containing an I-type immunity gene as a remnant of an earlier replacement of an I-type EI module) occurs, shifting back the 3′-end of the E-type effector and creating an array containing six different immunity genes.

Two theoretical alternatives to the successive replacement of an effector gene by a novel EI module could also explain the presence of immunity gene arrays. The first is the complete replacement of an EI-module by a DNA fragment encoding a different module containing a 3′-extended region with additional immunity genes. Although we found several examples of this type of event, they do not explain how the more complex immunity arrays initially formed, only how they were introduced in a new strain. Another alternative explanation for the existence of immunity gene arrays is that they are created by successive gene duplications. If duplication of immunity genes occurred, multiple homologous genes found in one strain would be more closely related to each other than to those found in other strains. However, as the phylogenies of immunity genes in Figs S5–S7 show, multiple alleles found in the a single genome do not cluster together and thus likely originate from different *Vibrio* strains rather than from duplication events. Furthermore, duplication events cannot explain heterogeneous arrays consisting of different immunity type genes.

Integration of DNA elements into chromosomal T6SS clusters of *Vibrio* thus likely occurs through at least two different mechanisms: I) normal homologous recombination leading to the replacement of a region in the T6SS cluster (as shown *in-vivo* by Koskiniemi *et al*.^43^ in *Salmonella*, where an orphan EI module replaces the variable 3′ region and immunity protein of a contact dependent toxin); and II) replacement of an effector by a novel EI module (perhaps) through homology-facilitated illegitimate recombination with conservation of the ancestral immunity gene. The latter was also hypothesized for the diversification of recombination hot spot (Rhs) protein coding loci (which includes T6SS regions) with constant 5′ and variable 3′ regions to explain the frequent observation of strings of “orphaned” 3′ regions^44^,^45^,^46^.

It appears likely that the first mechanism would occur more often, as closely related strains with different EI modules mostly do not contain orphan immunity genes indicative of illegitimate displacement events. For example, strains V52 and 2012EL-1759 are closely related and contain complete A- and I-type EI modules in the main cluster, respectively, with no orphan immunity genes in either of them (Fig. 2). Furthermore, experimental evidence in other organisms shows that homology-facilitated illegitimate recombination is quite rare (several orders of magnitude less frequent than regular homologous recombination in *Pseudomonas*^42^, *Streptococcus*^41^ and *Acinetobacter*^47^). While no comparisons of these two processes have been done for *Vibrio*, high rates of homologous recombination are commonly observed in multi-locus sequence typing or whole genome studies of this genus^48^,^49^,^50^,^51^,^52^. The insertion of T6SS EI pairs is reminiscent of site-specific recombination in the *Vibrio* chromosomal integron region^53^,^54^. In integrons, gene cassettes are added at an insertion site downstream of an integrase gene, whose gene product facilitates this process. Addition of a new gene cassette leads to the displacement of old gene cassettes to the back of the array^55^, which parallels our observation of immunity gene displacement. However, so far there exists no evidence that EI insertion is facilitated by an integrase in a similar manner. The relative uniformity of the O1/O139 T6SS arrays also stands in contrast with the variability in integron cassette content of that clade^56^, indicating that EI change, especially through illegitimate recombination, proceeds much less rapidly.

### The T6SS effector-immunity gene combination of pandemic *V. cholerae* strains evolved and spread through a series of horizontal gene transfer events

**Figure 5:**
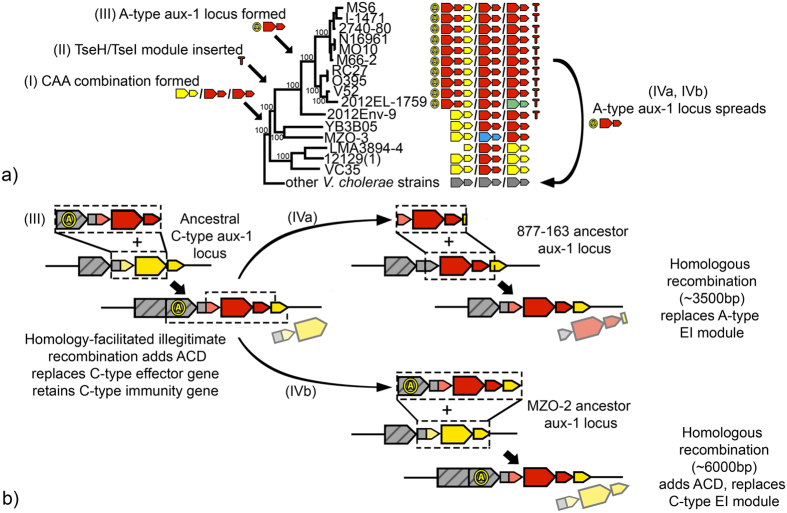
Evolution of the *Vibrio cholerae* aux-1 cluster. (**a**) Evolutionary events along the phylogeny of the lineage containing modern pandemic group *V. cholerae*. (**b**) Schematic representation of HGT events in the aux-1 cluster. (I) CAA module combination forms through homologous replacement of other EI modules in the ancestor of pandemic group *V. cholerae* and related strains. (II) TseH-TseI module is inserted close to the chromosomal integron region of the strain ancestral to the pandemic and pandemic sister group (represented by 2012-Env9). (III) Original C-type EI module is replaced by an A-type EI module in the ancestor of lineage containing pandemic *V. cholerae*, retaining the C-type immunity gene. Parts of the modern aux-1 cluster of pandemic *V. cholerae* are subsequently inserted into 877-163 (IVa) and MZO-2 (IVb) (see also Fig. S8). Arrows indicate coding sequences, lines noncoding regions. Striped arrows denote VgrG effectors; non-striped arrows variable effector (large arrows) or immunity genes (small arrows). Colours indicate homology between either effector or immunity genes. Identically coloured effector and immunity genes are part of the same EI module. Grey arrows indicate conserved genes. Regions of recombination are indicated by striped boxes.

Pandemic *V. cholerae* strains not only possess the ability to cause lethal disease in humans, but also a unique composition of T6SS modules that gives them (at least *in-vivo*) unmatched competitive abilities in interactions with conspecific strains^17^. All sequenced genomes of strains from the lineage containing pandemic *V. cholerae* (with the single known exception being the aforementioned 2012EL-1759) harbour the same T6SS A-type aux-1 module accompanied by an actin-crosslinking VgrG-1 and an extended 3′-region with a C-type immunity protein; A-type EI modules in aux-2 and the large cluster; and a TseH/TsiH module encoded close to the chromosomal integron region (Fig. 2). The specific combination of EI modules found at the three T6SS clusters of pandemic *V. cholerae* strains appears to have assembled progressively through a combination of both previously proposed integration mechanisms of horizontally transferred T6SS elements (Fig. 5a). (I) The CAA module combination, which is found in strains basal to the lineage that gave rise to pandemic *V. cholerae*, was likely the starting point for the evolution of the modern pandemic T6SS structure. (II) The antibacterial TseH-TseI module was likely acquired by the CAA-containing common ancestor of the lineage encompassing pandemic strains and its sister group (exemplified by 2012-Env9), potentially providing a competitive advantage over strains lacking this genetic element^20^. (III) The A-type EI module subsequently replaced the ancestral C-type effector while displacing the C-type immunity gene into the extended 3′-region in the ancestor of the lineage that gave rise to modern pandemic strains (Fig. 5b). A single gene phylogeny of the C-type immunity gene provides some evidence for this hypothesis, as the alleles found in the pandemic strains lineage and its sister group strains remain comparatively similar, in congruence with the common ancestry of these groups (Fig. S4). Acquisition of a new, rare A-type EI module while retaining the C-type immunity gene (whose expression is up-regulated nearly 2 folds during infection of the host^14^) likely enhanced the competitive advantage of strains in the pandemic lineage. Whether the VgrG-1 actin crosslinking domain shared by all *V. cholerae* in the lineage containing pandemic strains was included in that recombination event or inserted at a separate point before or after remains unclear, although parsimony would imply integration in a single event.

Interestingly, parts of the lethal T6SS structure have proceeded to spread from the lineage containing pandemic strains into distantly related, non-pandemic strains. Both strains MZO-2 and 877-163 possess aux-1 clusters containing effector and immunity genes nearly identical to their homologs in pandemic strains (Figs S4 and S5), making it likely that the latter was the donor. In accordance to our finding that integration of recombinant DNA into T6SS clusters does not occur at specific sites, the size of recombined regions differs for both strains (Figs 5b and S8). (IVa) The size of the region received by 877-163 is around 3.5 Kbp and includes the 3′-end of *tap-1* upstream of the EI module and a small part of the C-type immunity gene downstream. (IVb) In contrast, the region received by MZO-2 extends beyond the adaptor *tap-1* gene and the actin-crosslinking domain of *vgrG-1* upstream of the EI module and beyond the C-type immunity gene downstream (more than 6 Kbp). We interpret this as evidence that, similar to Rhs elements in other Gram negative bacteria^57^, complex E-I arrays created by successive displacements of effector genes by E-I modules could form a pool of structurally stable elements that can be transferred between diverse strains through frequent homologous recombination.

## Conclusions

Our observation of mosaic arrays of immunity genes in the T6SS clusters of *V. cholerae* and closely related species suggests a selective advantage for the presence of multiple immunity genes. The retention of immunity genes by shifting them into the extended 3′-regions of the *Vibrio* T6SS clusters provides a single cell within an otherwise homogeneous population a mechanism to successfully acquire a EI module without being killed by surrounding kin bacteria. *V. cholerae* becomes naturally competent when reaching high cell densities on chitinous surfaces^58^, conditions that also lead to up-regulation of T6SS gene expression^7^. Therefore, any cell acquiring a new EI module is probably surrounded by now incompatible sister cells. Since even more effective EI module combinations succumb to less effective ones when greatly outnumbered^17^, a newly acquired EI module combination would likely be rapidly overwhelmed by sister cells. Our observation that the extended 3′-region of T6SS clusters retains ORFs coding for additional immunity proteins provides an explanation for the EI diversity reported previously and validated here. EI modules could be successfully acquired in a numerically superior population of incompatible cells by only replacing the effector, but retaining the immunity gene. Due to the aforementioned simultaneous upregulation of T6SS activity and natural competence, incoming non-compatible cells killed by the T6SS of resident Vibrios would represent an easily available source of DNA encoding new EI modules and other potentially beneficial genetic elements^7^. Larger amounts of DNA freed from subsequently killed former sister cells could then provide a readily available additional food source^59^.

After acquisition of a novel EI module by a lineage of bacteria, expression of additional immunity proteins (such as the C-type immunity protein VC1420 in pandemic strains^14^) could also confer protection against more distantly related strains with different effectors. Additional immunity proteins encoded in the 3′-extended region belonging to the same type as the main immunity protein present in the EI module, but displaying some sequence divergence could protect strains against similarly divergent effectors that cannot be effectively bound by the main immunity protein^36^. This would be particularly beneficial in competition involving widespread and diverse EI modules such as the aux-1 C-type.

Relaxed selective pressure on a redundant immunity gene could furthermore give cells a significant edge in a T6SS mediated arms race. In colicin E-I modules, mutations in an immunity gene conferring additional resistance to foreign effector types are thought to be followed by mutations in effector genes that enable it to avoid immunity of other strains, leading to the emergence of a competitively superior strain^40^. A second copy of an immunity gene would allow one of the genes to diverge without having to retain the immunity function against the cell’s own effector and could considerably speed up the evolution of novel functionalities^45^. Furthermore, it would appear mechanistically easier for a single effector to mutate to overcome binding by immunity proteins of other strains than for a single immunity protein being able to bind all potential effector variants. A larger repertoire of immunity proteins, even of the same type, could thus confer an advantage in an effector-rich environment.

Thus, understanding compatibility of various *Vibrio* strains might require taking into account not only the effectors and immunity proteins encoded in EI modules, but also those found in the extended 3′-regions of T6SS clusters, as well as sequence divergence within effector and immunity proteins of the same type.

In summary, we provide a comprehensive overview of the *V. cholerae* T6SS EI module diversity in a phylogenetic context, expand the repertoire of the *Vibrio* T6SS by multiple novel putative effectors, extend T6SS clusters to include an additional 3′ region, and put forth a hypothetical model for the evolution of mosaic immunity gene arrays in this 3′ extended region. Furthermore, our analysis makes it possible to trace the genesis of possibly the most effective module combination, found in the *V. cholerae* lineage containing pandemic strains, through stepwise acquisition of singular elements along their pathway from harmless environmental bacteria to deadly human pathogens.

## Additional Information

**How to cite this article:** Kirchberger, P. C. *et al*. Sequential displacement of Type VI Secretion System effector genes leads to evolution of diverse immunity gene arrays in *Vibrio cholerae. Sci. Rep.* **7**, 45133; doi: 10.1038/srep45133 (2017).

**Publisher's note:** Springer Nature remains neutral with regard to jurisdictional claims in published maps and institutional affiliations.

## Supplementary Material

Supplementary Figures and Tables
