# Supplementary material for: Sequential displacement of Type VI Secretion System effector genes leads to evolution of diverse immunity gene arrays in Vibrio cholerae
Source: Sci Rep. 2017 Mar 22;7:45133. doi: 10.1038/srep45133 (PMC5361080; doi:10.1038/srep45133)
Supplement: Supplementary Figures and Tables [file srep45133-s1.pdf]

Supplementary information

Sequential displacement of Type VI Secretion System effector genes leads to evolution of diverse immunity gene arrays in *Vibrio cholerae*

Paul C. Kirchberger[1], Daniel Unterweger[2], Daniele Provenzano[3], Stefan Pukatzki[4] and Yan Boucher[1#]

[1]Department of Biological Sciences, University of Alberta, Alberta, Canada

[2] Department of Zoology, University of Oxford, Oxford, United Kingdom

[3] Department of Biological Sciences, University of Texas Rio Grande Valley, Texas, United States

[4] Department of Immunology & Microbiology, University of Colorado School of Medicine, Colorado, United States

#Corresponding author: Yan Boucher, Department of Biological Sciences,

University of Alberta, Edmonton, AB T6G 2E9, Canada; yboucher@ualberta.ca

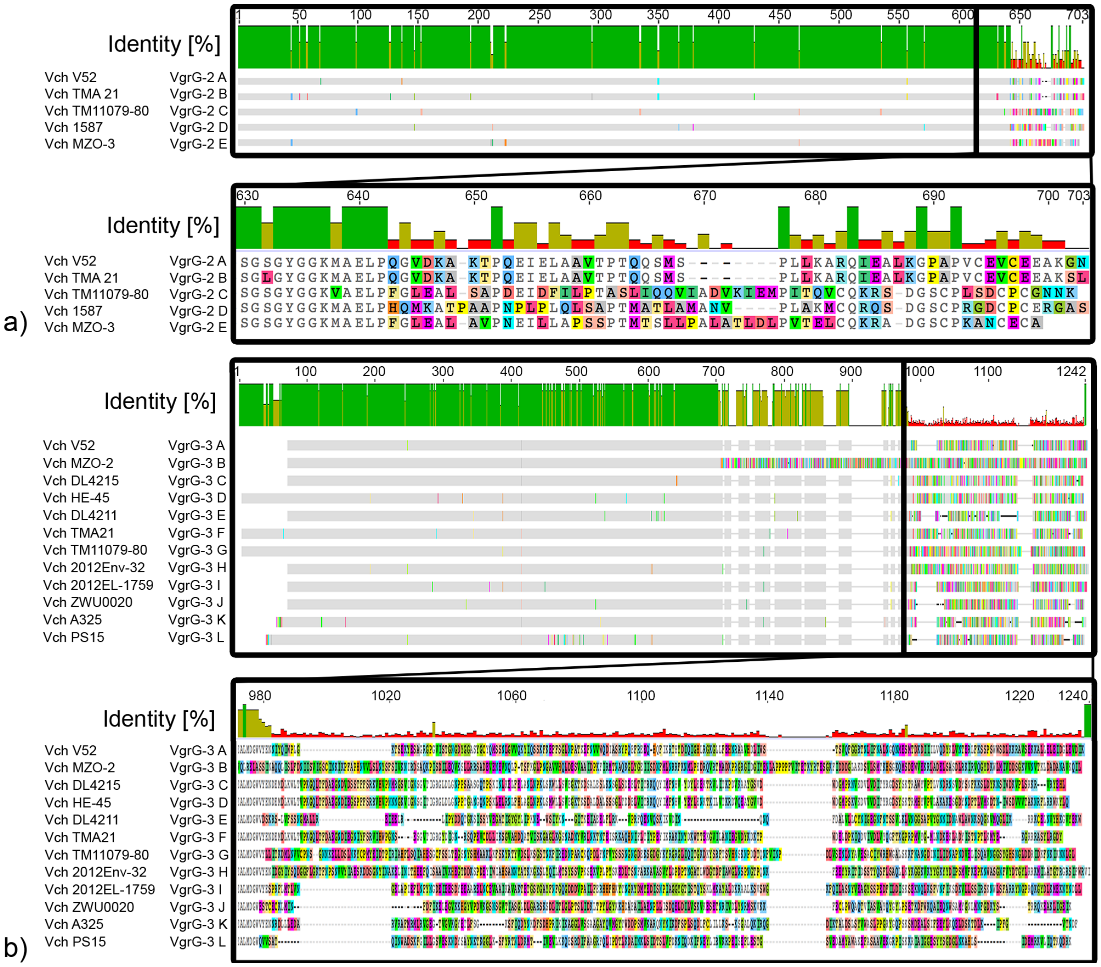

Figure S1: Amino acid alignment of VgrG-2 and VgrG-3. a) Alignment of VgrG-2 proteins found in aux-2 cluster encoding different types of cargo effectors b) Alignment of VgrG-3 proteins in large cluster encoding different variable C-terminal regions. Conserved sites are depicted as grey, while colour indicates amino acid change compared to the consensus. Variable C-terminal regions of VgrG-2 and VgrG-3 are enlarged. Vch = *Vibrio cholerae.*

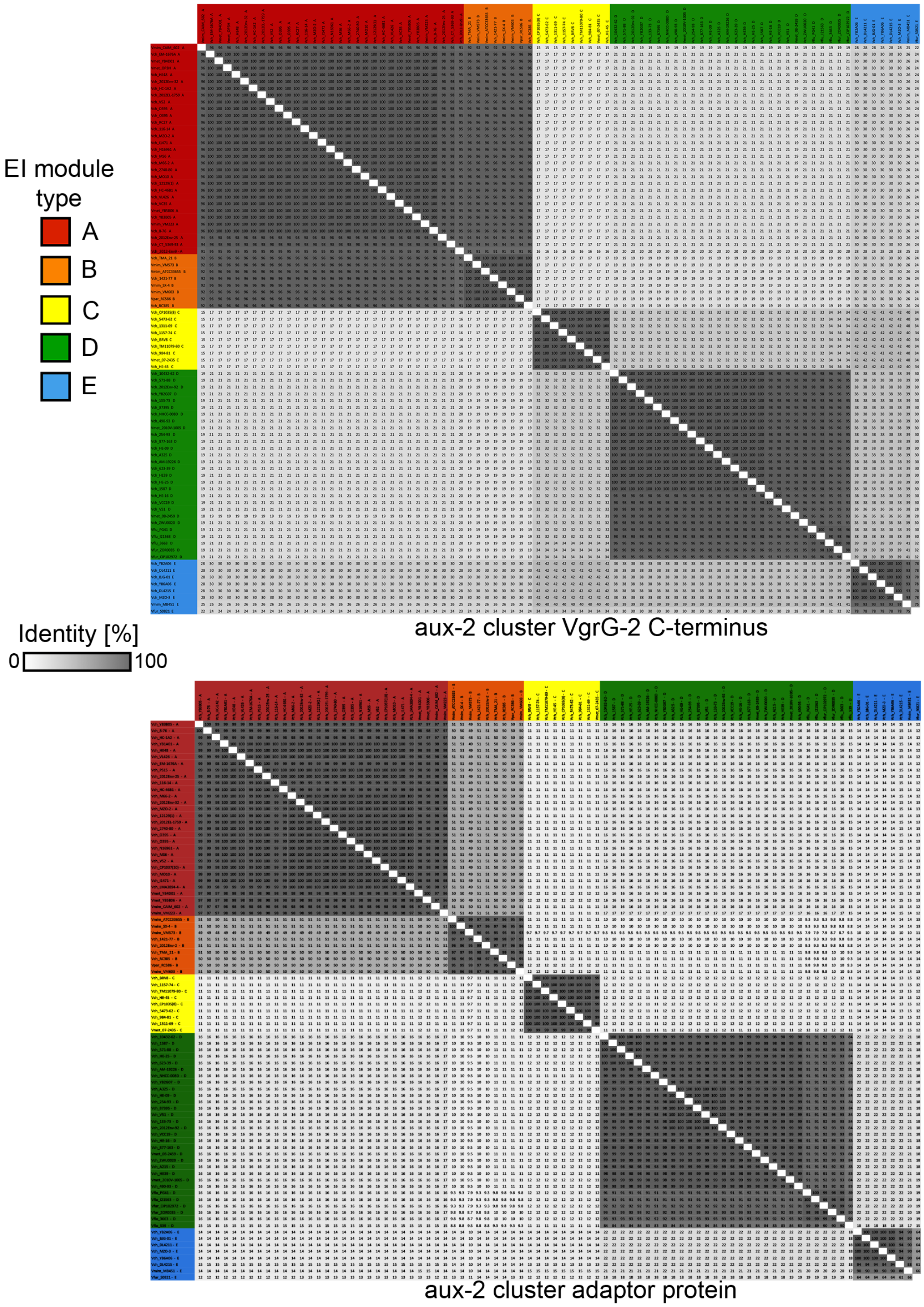

Figure S2: Amino acid identity of variable region of VgrG-2 and aux-2 adaptor proteins. Heat maps indicate percent identity of aligned protein sequences found in investigated strains. Colouring of strain names indicates type of EI module present

in the aux-2 cluster of the respective strain. Pseudogenes with interrupted reading frames were not included in the comparison. Vch = *Vibrio cholerae,* Vmet = *V. metoecus*, Vmim = *V. mimicus*, Vfur = *V. furnissii*, Vflu = *V. fluvialis*, Vpar = *V. sp.* RC586*.*

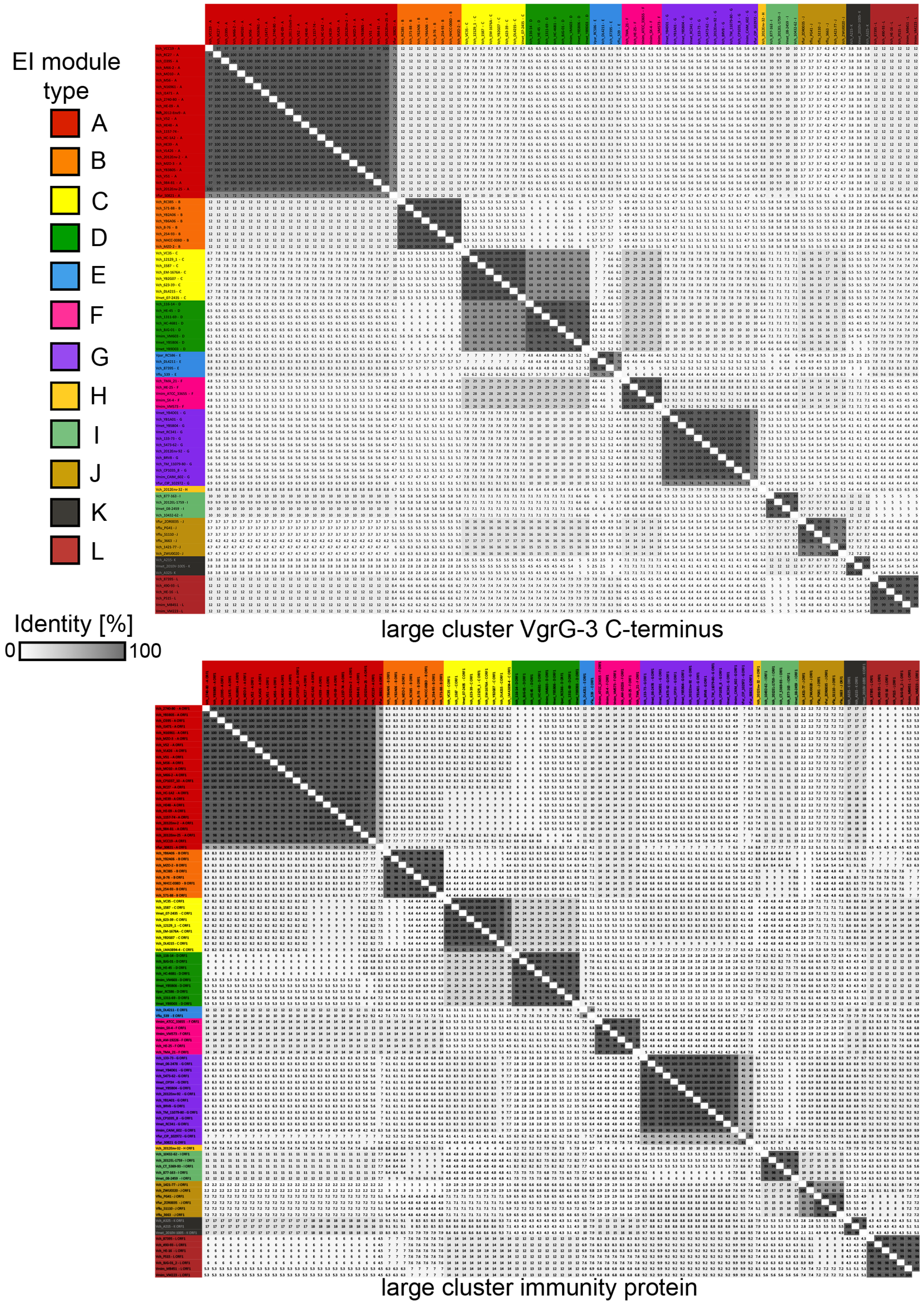

Figure S3: Amino acid identity of variable region of VgrG-3 and cognate immunity gene. Heat maps indicate percent identity of proteins found in investigated strains. Colouring of strain names indicates type of EI module present

in the large cluster of the respective strain. Pseudogenes with interrupted reading frames were not included in the comparison. Vch = *Vibrio cholerae,* Vmet = *V. metoecus*, Vmim = *V. mimicus*, Vfur = *V. furnissii*, Vflu = *V. fluvialis*, Vpar = *V. sp.* RC586*.*

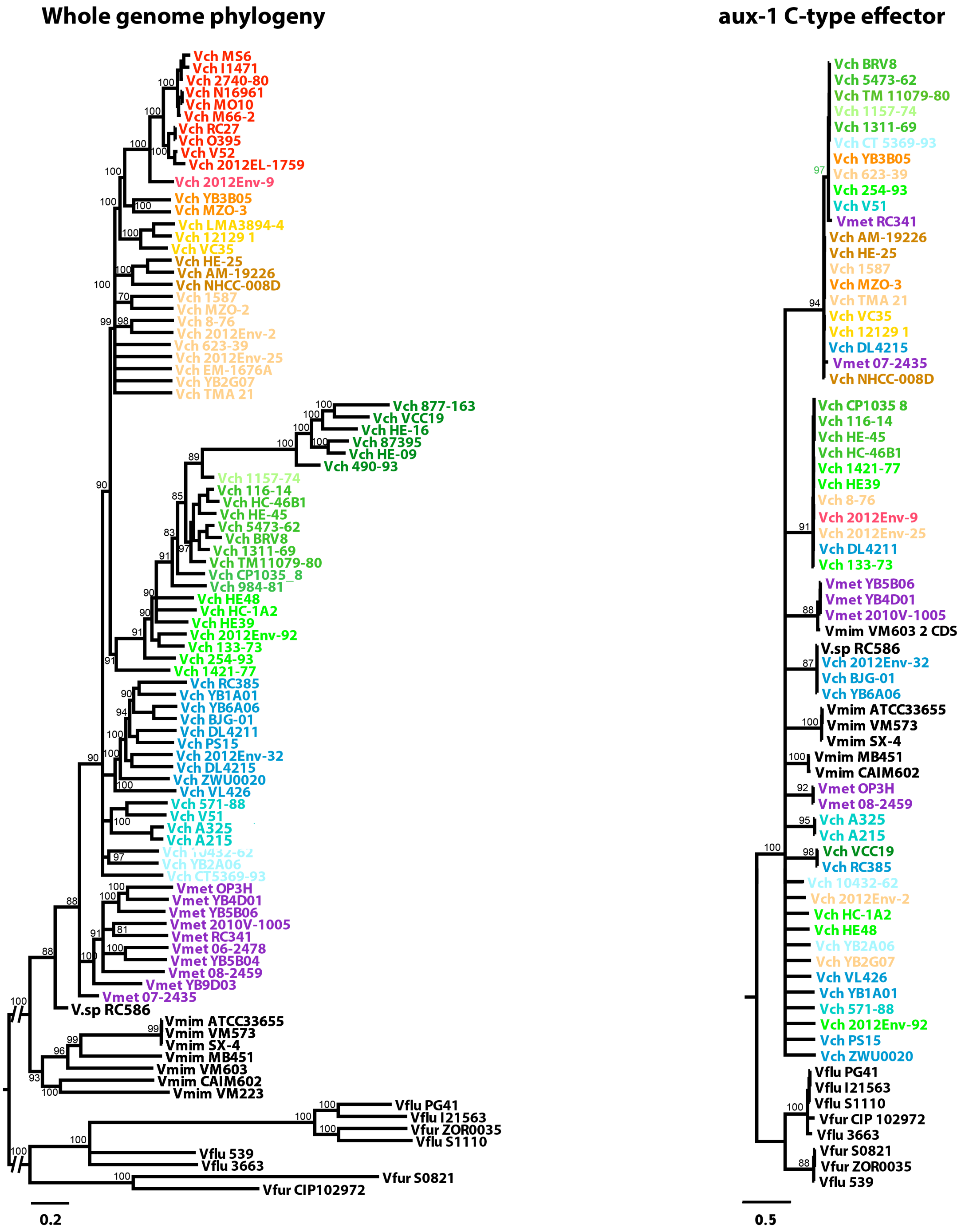

Figure S4: Incongruence between whole genome phylogeny and single gene phylogeny of aux-1 C-type effector. Whole genome tree corresponds to Figure 2. Related strains are colored similarly to ease comparison between tree topologies

but do not necessarily correspond to monophyletic groups. Aux-1 C-type effector phylogeny was calculated using the GTR+Gamma Maximum Likelihood model implemented in RAxML based a 2,315bp alignment. Black dots indicate orphan immunity genes (i.e. genes not directly downstream of an effector). Statistical branch support was obtained from 100 bootstrap repeats. Branches with support <70 were collapsed, as such every branch has a bootstrap support of >70. Scale bar indicates substitutions/site. Double dashes through branches indicates cut by length of 4.0 substitutions/site. Vch = *Vibrio cholerae,* Vmet = *V. metoecus*, Vmim = *V. mimicus*, Vfur = *V. furnissii*, Vflu = *V. fluvialis*.

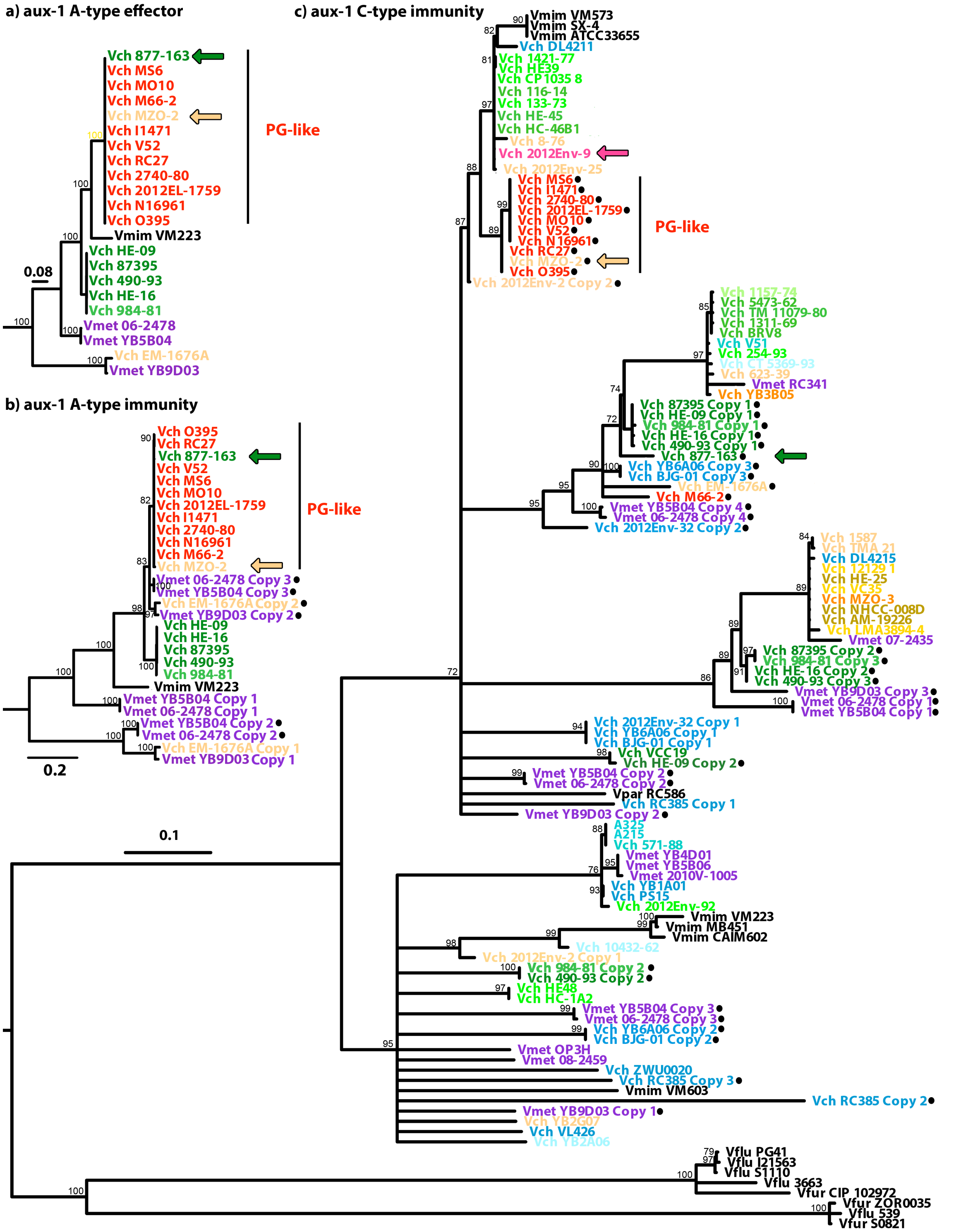

Figure S5: Single gene phylogenies of aux-1 a) A-type effector, b) A-type immunity and c) C-type immunity genes. Colouring of strains corresponds to whole genome tree of figure S4. Arrows indicate strains of interest discussed in text.

Phylogenetic trees were calculated using the GTR+Gamma Maximum Likelihood model implemented in RAxML based on single gene alignments (aux-1 A-type effector: 2,040bp; aux-1 A-type immunity: 754bp, aux-1 C-type immunity: 740bp. Black dots indicate orphan immunity genes (i.e. genes not directly downstream of an effector). Statistical branch support was obtained from 100 bootstrap repeats. Branches with support <70 were collapsed, as such every branch has a bootstrap support of >70. Scale bar indicates substitutions/site. Vch = *Vibrio cholerae,* Vmet = *V. metoecus*, Vmim = *V. mimicus*, Vfur = *V. furnissii*, Vflu = *V. fluvialis*.

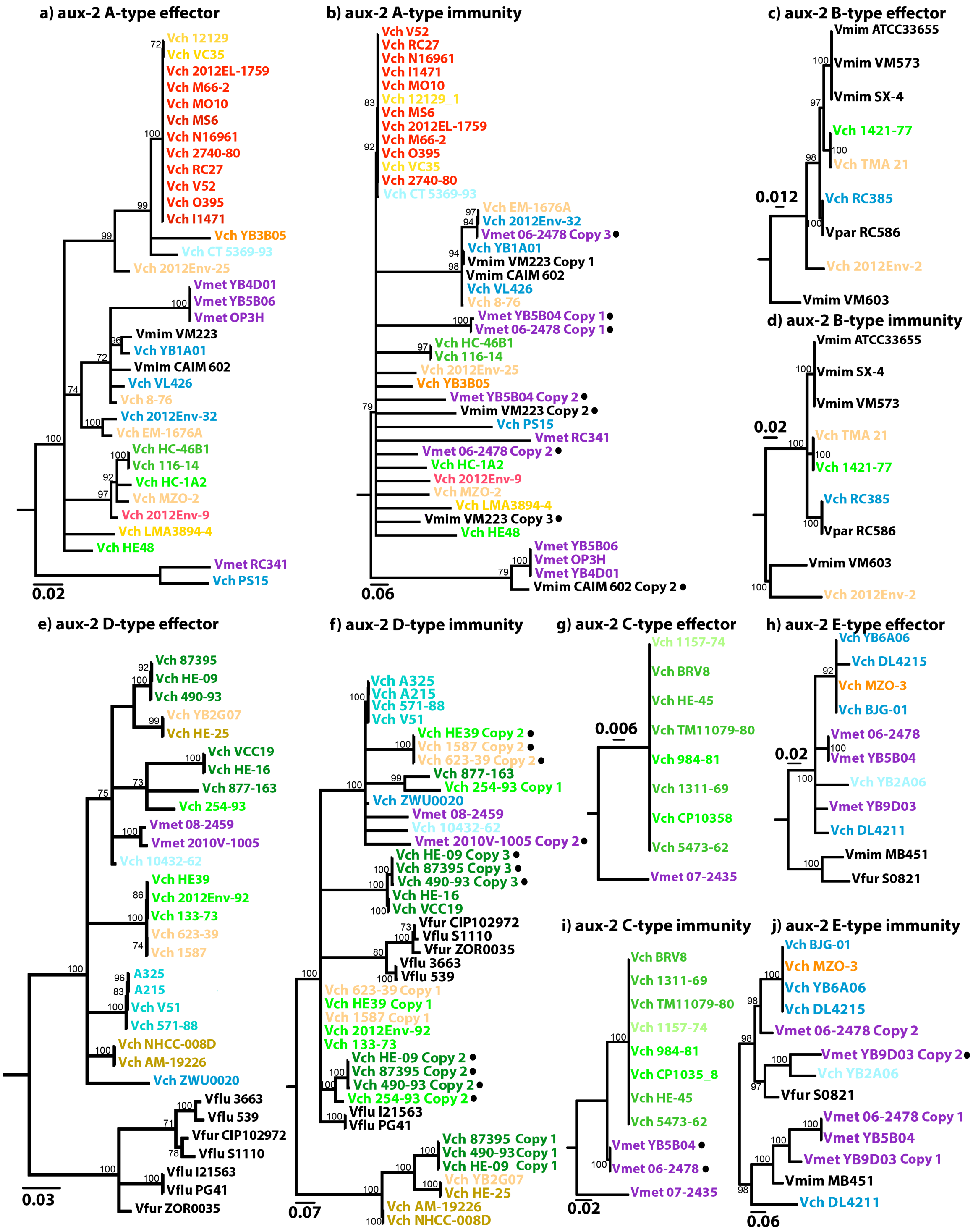

Figure S6: Single gene phylogenies of aux-2 effector and immunity genes. a) A-type effector b) A-type immunity c) B-type effector d) B-type immunity e) D-type effector f) D-type immunity g) C-type effector h) E-type effector i) C-type immunity

j) E-type immunity. Colouring of strains corresponds to whole genome tree of figure S4. Phylogenetic trees were calculated using the GTR+Gamma Maximum Likelihood model implemented in RAxML based on single gene alignments (aux-2 A-type effector: 3,268 bp; aux-2 B-type effector: 3,261 bp; aux-2 C-type effector: 3,531 bp; aux-2 D-type effector: 3,726 bp; aux-2 E-type effector: 3,594 bp; aux-2 A-type immunity: 741 bp; aux-2 B-type immunity: 1,119 bp; aux-2 C-type immunity: 951bp; aux-2 D-type immunity: 1,162 bp; aux-2 E-type immunity: 1,018 bp. Black dots indicate orphan immunity genes (i.e. genes not directly downstream of an effector). Statistical branch support was obtained from 100 bootstrap repeats. Branches with support <70 were collapsed, as such every branch has a bootstrap support of >70. Scale bar indicates substitutions/site. Vch = *Vibrio cholerae,* Vmet = *V. metoecus*, Vmim = *V. mimicus*, Vfur = *V. furnissii*, Vflu = *V. fluvialis*.

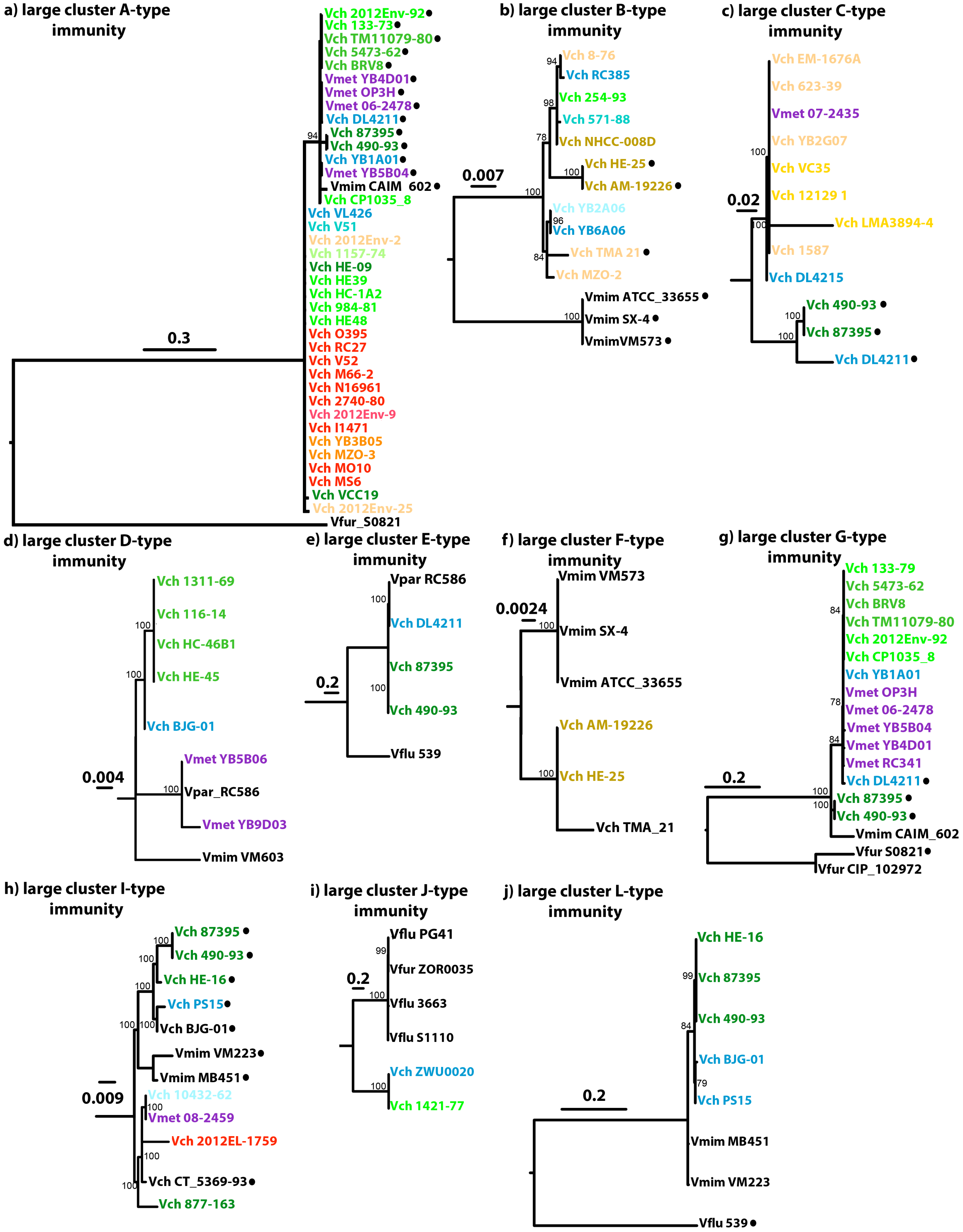

Figure S7: Single gene phylogenies of large cluster immunity genes. Colouring of strains corresponds to whole genome tree of figure S4. Phylogenetic trees were calculated using the GTR+Gamma Maximum Likelihood model implemented in

RAxML based on single gene alignments (main A-type immunity: 402 bp; main B-type immunity: 966 bp; main C-type immunity: 384bp; main D-type immunity: 399 bp; main E-type immunity: 346 bp; main F-type immunity: 409 bp; main G-type immunity: 585; main I-type immunity: 445 bp; main J-type immunity: 384 bp; main L-type immunity: 402 bp. Black dots indicate orphan immunity genes (i.e. genes not directly downstream of an effector). Statistical branch support was obtained from 100 bootstrap repeats. Branches with support <70 were collapsed, as such every branch has a bootstrap support of >70. Scale bar indicates substitutions/site. Vch = *Vibrio cholerae,* Vmet = *V. metoecus*, Vmim = *V. mimicus*, Vfur = *V. furnissii*, Vflu = *V. fluvialis*.

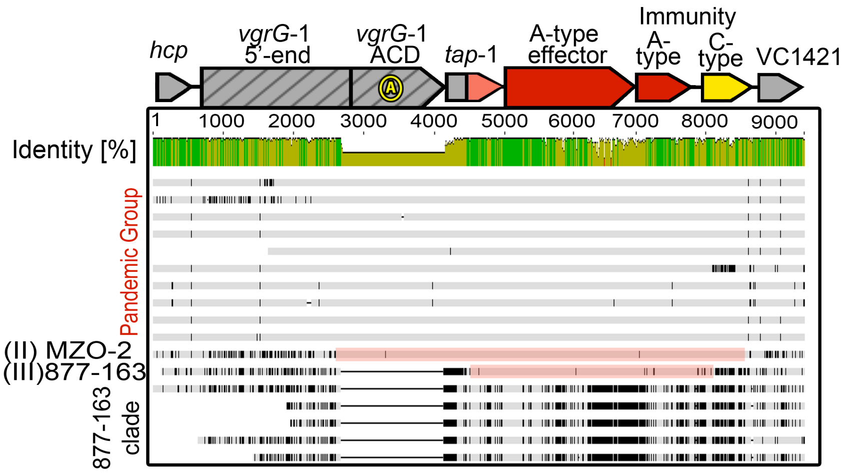

Figure S8: Nucleotide alignment of aux-1 clusters of the lineage containing pandemic *V. cholerae* and putative recombinant regions. Grey regions indicated conserved sites while black lines indicate divergence from the consensus sequence. Red bars highlight regions in MZO-2 and 877-163 with high identity to the pandemic group aux-1 cluster, indicating putative horizontal gene transfer.

Supplementary Table 1: Accession numbers of genomes used in this study.

Species	Strain	Accession	Accession 2
V. cholerae	MS6	AP014524.1	AP014525.1
V. cholerae	I-1471	CM003111.1	CM003112.1
V. cholerae	2740-80	AAUT00000000.1	
V. cholerae	N16961	AE003852.1	AE003853.1
V. cholerae	MO10	AAKF00000000.3	
V. cholerae	M66-2	CP001233.1	CP001234.1
V. cholerae	RC27	ADAI00000000.1	
V. cholerae	O395	CP001235.1	CP001236.1
V. cholerae	V52	AAKJ00000000.2	
V. cholerae	2012EL-1759	JNEW00000000.1	
V. cholerae	2012Env-9	JSTH00000000.1	
V. cholerae	CP1037(10)	ALDB00000000.1	
V. cholerae	YB3B05	LBGB00000000.1	
V. cholerae	MZO-3	AAUU00000000.1	
V. cholerae	LMA3984-4	CP002555.1	CP002556.1
V. cholerae	12129(1)	ACFQ00000000.1	
V. cholerae	VC35	AMBR00000000.1	
V. cholerae	HE-25	ALEC00000000.1	
V. cholerae	AM-19226	AATY00000000.1	
V. cholerae	NHCC-008D	APGC00000000.1	
V. cholerae	1587	AAUR00000000.1	
V. cholerae	MZO-2	AAWF00000000.1	
V. cholerae	8-76	JIDN00000000.1	
V. cholerae	2012Env-2	JSTD00000000.1	
V. cholerae	623-39	AAWG00000000.1	
V. cholerae	2012Env-25	JSTE00000000.1	
V. cholerae	EM-1676A	APFY00000000.1	
V. cholerae	YB2G07	LBGA00000000.1	
V. cholerae	TMA21	ACHY00000000.1	
V. cholerae	1421-77	JMBL00000000.1	
V. cholerae	254-93	JMBP00000000.1	
V. cholerae	133-73	JIDK00000000.1	
V. cholerae	2012Env-92	JSTJ00000000.1	
V. cholerae	HE-39	AFOQ00000000.1	
V. cholerae	HC-1A2	AJRO00000000.1	
V. cholerae	HE-48	AFOR00000000.1	
V. cholerae	984-81	JMBM00000000.1	
V. cholerae	CP1035(8)	AJRM00000000.1	
V. cholerae	TMM11079-80	ACHW00000000.1	

V. cholerae	1311-69	JIDJ00000000.1
V. cholerae	BRV8	CTBD00000000.1
V. cholerae	5473-62	JIDI00000000.1
V. cholerae	HE-45	ALED00000000.1
V. cholerae	HC-46B1	AJSL00000000.1
V. cholerae	116-14	CGHE00000000.1
V. cholerae	1157-74	JIDL00000000.1
V. cholerae	490-93	JIDQ00000000.1
V. cholerae	HE-09	AFOP00000000.1
V. cholerae	87395	APFL00000000.1
V. cholerae	HE-16	ALEB00000000.1
V. cholerae	VCC19	ATEV00000000.2
V. cholerae	877-163	LBNV00000000.1
V. cholerae	RC385	AAKH00000000.3
V. cholerae	YB1A01	LBCL00000000.1
V. cholerae	YB6A06	LBGK00000000.1
V. cholerae	BJGO1	AFOU00000000.1
V. cholerae	DL4211	MOLL00000000.1
V. cholerae	PS15	AIJR00000000.1
V. cholerae	2012Env-32	JSTF00000000.1
V. cholerae	DL4215	MOLM00000000.1
V. cholerae	ZWU0020	JRJX00000000.1
V. cholerae	VL426	ACHV00000000.1
V. cholerae	571-88	JIDO00000000.1
V. cholerae	V51	AAKI00000000.2
V. cholerae	A325	CWSO00000000.1
V. cholerae	A215	CWSL00000000.1
V. cholerae	10432-62	GCA_000969265.1
V. cholerae	YB2A06	LBFX00000000.1
V. cholerae	CT5369-93	ADAL00000000.1
V. metoecus	OP3H	JJMN00000000.1
V. metoecus	YB4D01	LBGO00000000.1
V. metoecus	YB5B06	LBGQ00000000.1
V. metoecus	2010V-1005	LCUG00000000.1
V. metoecus	RC341	ACZT00000000.1
V. metoecus	06-2478	LCUD00000000.1
V. metoecus	YB5B04	LBGP00000000.1
V. metoecus	08-2459	LCUF00000000.1
V. metoecus	YB9D03	LBGR00000000.1
V. metoecus	07-2435	LCUE00000000.1
V. mimicus	VM223	ADAJ00000000.1
V. mimicus	CAIM602	AOMO00000000.1
V. mimicus	VM603	ACYU00000000.1

V. mimicus	MB451	ADAF00000000.1	
V. mimicus	SX-4	ADOO00000000.1	
V. mimicus	VM573-73	ACYV00000000.1	
V. mimicus	ATCC33655	NZ_CP014042.1	NZ_CP014043.1
V. furnissii	CIP102972	ACZP00000000.1	
V. furnissii	SO821	LKHS00000000.1	
V. fluvialis	3663	JXXQ00000000.1	
V. fluvialis	539	JQHX00000000.1	
V. fluvialis	S1110	LKHR00000000.1	
V. furnissii	ZOR0035	JTLJ00000000.1	
V. fluvialis	I21563	ASXT00000000.1	
V. fluvialis	PG41	ASXS00000000.1	

Supplementary Table 2: Genomic locations of genomic cluster of interest

Strain	cluster	gene	Sequence ID	start	end
YB5B04	aux-1	A-type effector	LBGP01000007.1	187794	189711
	aux-1	A-type immunity gene 1	LBGP01000007.1	187081	187797
	aux-1	A-type immunity gene 2	LBGP01000007.1	186365	187072
	aux-1	A-type immunity gene 3	LBGP01000007.1	185622	186254
	aux-1	C-type immunity gene 1	LBGP01000007.1	184744	185481
	aux-1	C-type immunity gene 2	LBGP01000007.1	183984	184769
	aux-1	C-type immunity gene 3	LBGP01000007.1	183227	184009
	aux-1	C-type immunity gene 4	LBGP01000007.1	182467	183252
	aux-1	threonine efflux pump (VCA1421)	LBGP01000007.1	181741	182379
06-2478	aux-1	A-type effector	LCUD01000037.1	2973	4887
	aux-1	A-type immunity gene 1	LCUD01000037.1	4884	5603
	aux-1	A-type immunity gene 2	LCUD01000037.1	5612	6238
	aux-1	A-type immunity gene 3	LCUD01000037.1	6431	7063
	aux-1	C-type immunity gene 1	LCUD01000037.1	7204	7941
	aux-1	C-type immunity gene 2	LCUD01000037.1	7916	8701
	aux-1	C-type immunity gene 3	LCUD01000037.1	8676	9458
	aux-1	C-type immunity gene 4	LCUD01000037.1	9433	10065
	aux-1	threonine efflux pump (VCA1421)	LCUD01000037.1	10310	10948
LMA3894-4	aux-1	vgrG-1	NC_017270.1	1411393	1412703
	aux-1	vgrG-1/C-type immunity fusion	NC_017270.1	1412631	1414052
YB5B04	aux-2	dehydrogenase	LBGP01000034.1	3102	4103

	aux-2	IS5 family transposase	LBGP01000034.1	1559	2479
	aux-2	aux-2 C-type immunity (partial)	LBGP01000034.1	902	1348
	aux-2	aux-2 A-type immunity 1	LBGP01000034.1	1	721
	aux-2	aux-2 A-type immunity 2	LBGP01000005.1	411	1133
	aux-2	GST (VCA0022)	LBGP01000005.1	1242	1643
06-2478	aux-2	dehydrogenase	LCUD01000013.1	74065	75066
	aux-2	IS5 family transposase	LCUD01000013.1	72522	73442
	aux-2	aux-2 C-type immunity (partial)	LCUD01000013.1	71865	72311
	aux-2	aux-2 A-type immunity 1	LCUD01000013.1	70785	71684
	aux-2	aux-2 A-type immunity 2	LCUD01000013.1	69764	70486
	aux-2	aux-2 A-type immunity 3	LCUD01000013.1	68731	69465
	aux-2	GST (VCA0022)	LCUD01000013.1	68227	68628
V52	large cluster	vgrG-3 A-type	MIPN01000011.1	115720	118773
	large cluster	large cluster A-type immunity	MIPN01000011.1	118770	119138
	large cluster	rbsD (VCA0125)	MIPN01000011.1	119779	120249
S0821	large cluster	vgrG-3 A-type	LKHS01000001.1	189108	192227
	large cluster	large cluster A-type immunity	LKHS01000001.1	192224	192592
	large cluster	large cluster G-type immunity	LKHS01000001.1	192600	193178
	large cluster	rbsD (VCA0125)	LKHS01000001.1	193953	194372
MZO-2	large cluster	vgrG-3 B-type (C-terminus)	MIKJ01000102.1	118909	120225
	large cluster	large cluster B-type immunity	MIKJ01000102.1	120246	121211
	large cluster	rbsD (VCA0125)	MIKJ01000102.1	121752	122222
DL4215	large cluster	vgrG-3 C-type	KF228946.1	29404	32517
	large cluster	large cluster C-type immunity	KF228946.1	32504	32887
	large cluster	rbsD (VCA0125)	KF228946.1	33758	34228
HE-45	large cluster	vgrG-3 D-type	ALED01000027.1	175382	178705
	large cluster	large cluster D-type immunity	ALED01000027.1	175002	175400
	large cluster	large cluster D-type ORF2	ALED01000027.1	174526	174972
	large cluster	rbsD (VCA0125)	ALED01000027.1	173520	173990

DL4211	large cluster	vgrG-3 E-type	KF228945.1	24923	27853
	large cluster	large cluster E-type immunity	KF228945.1	27847	28167
	large cluster	large cluster C-type immunity	KF228945.1	28225	28608
	large cluster	large cluster G-type immunity	KF228945.1	28825	29406
	large cluster	large cluster A-type immunity	KF228945.1	29372	29767
	large cluster	rbsD (VCA0125)	KF228945.1	30408	30878
RC586	large cluster	vgrG-3 E-type	ADBD01000008.1	513368	516298
	large cluster	large cluster E-type immunity	ADBD01000008.1	516292	516612
	large cluster	vgrG-3 D-type (partial)	ADBD01000008.1	516590	517276
	large cluster	large cluster D-type immunity	ADBD01000008.1	517258	517656
	large cluster	large cluster D-type ORF2	ADBD01000008.1	517686	518132
	large cluster	rbsD (VCA0125)	ADBD01000008.1	518666	519136
539	large cluster	vgrG-3 E-type	JQHX01000001.1	182203	185001
	large cluster	large cluster E-type immunity	JQHX01000001.1	181892	182209
	large cluster	large cluster L-type immunity	JQHX01000001.1	181294	181692
	large cluster	rbsD (VCA0125)	JQHX01000001.1	180043	180600
TMA 21	large cluster	vgrG-3 F-type	ACHY01000016.1	609284	612562
	large cluster	large cluster F-type immunity	ACHY01000016.1	612562	612969
	large cluster	large cluster B-type immunity	ACHY01000016.1	612997	613962
	large cluster	rbsD (VCA0125)	ACHY01000016.1	614503	614973
TM11079-80	large cluster	vgrG-3 G-type	ACHW01000028.1	35703	39056
	large cluster	large cluster G-type immunity	ACHW01000028.1	39061	39642
	large cluster	large cluster A-type immunity	ACHW01000028.1	39608	40003
	large cluster	rbsD (VCA0125)	ACHW01000028.1	40644	41114
2012Env-32	large cluster	vgrG-3 H-type (partial)	JSTF01000013.1	76141	78329
	large cluster	large cluster H-type immunity	JSTF01000013.1	75555	76139

	large cluster	rbsD (VCA0125)	JSTF01000013.1	74539	75009
2012EL-1759	large cluster	vgrG-3 I-type	JNEW01000035.1	475249	478314
	large cluster	large cluster I-type immunity	JNEW01000035.1	478301	478744
	large cluster	rbsD (VCA0125)	JNEW01000035.1	479308	479778
ZWU0020	large cluster	vgrG-3 J-type	JRJX01000133.1	80843	83827
	large cluster	large cluster J-type immunity	JRJX01000133.1	83836	84207
	large cluster	large cluster H-type immunity	JRJX01000133.1	84378	84683
	large cluster	rbsD (VCA0125)	JRJX01000133.1	85229	85699
PG41	large cluster	vgrG-3 J-type	ASXS01000008.1	176240	179278
	large cluster	large cluster J-type immunity	ASXS01000008.1	179275	179658
	large cluster	rbsD (VCA0125)	ASXS01000008.1	180443	180862
A325	large cluster	vgrG-3 K-type	CWSO01000002.1	410073	413039
	large cluster	large cluster K-type immunity	CWSO01000002.1	413017	413454
	large cluster	large cluster K-type ORF2	CWSO01000002.1	413460	413891
	large cluster	rbsD (VCA0125)	CWSO01000002.1	414493	414963
PS15	large cluster	vgrG-3 L-type	AIJR01000051.1	84155	87223
	large cluster	large cluster L-type immunity	AIJR01000051.1	87220	87624
	large cluster	large cluster I-type immunity	AIJR01000051.1	87649	88149
	large cluster	rbsD (VCA0125)	AIJR01000051.1	88713	89183
490-93	large cluster	vgrG-3 L-type	JIDQ01000041.1	5600	8575
	large cluster	large cluster L-type immunity	JIDQ01000041.1	8572	8976
	large cluster	large cluster I-type immunity	JIDQ01000041.1	9001	9501
	large cluster	vgrG-3 E-type (partial)	JIDQ01000041.1	9856	10855
	large cluster	large cluster E-type immunity	JIDQ01000041.1	10822	11169
	large cluster	large cluster C-type immunity	JIDQ01000041.1	11227	11610
	large cluster	large cluster G-type immunity	JIDQ01000041.1	11831	12412
	large	large cluster A-type immunity	JIDQ01000041.1	12378	12773

	cluster				
	large cluster	rbsD (VCA0125)	JIDQ01000041.1	13388	13858
